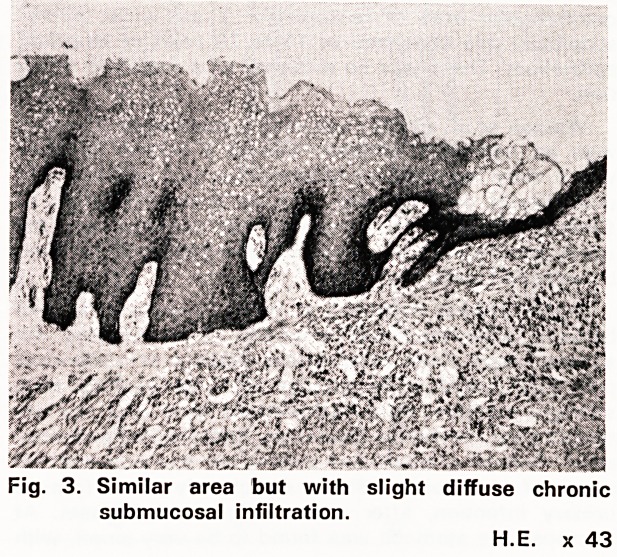# Gastric Leukoplakia Associated with Fundal Leiomyoma

**Published:** 1975

**Authors:** R. Salm

**Affiliations:** Department of Histopathology, Royal Cornwall Hospital (Treliske), Truro, Cornwall


### Gastric Leukoplakia Associated With Fundal Leiomyoma

By
**R. Salm**

Department of Histopathology, Royal Cornwall Hospital (Treliske), Truro, Cornwall

Islands of gastric mucosa often occur in the terminal, and less frequently in the cervical oesophagus (Stout and Lattes, 1957), but, conversely, the presence of heterotopic islands of oesophageal epithelium in the gastric fundus has been observed only occasionally (Boswell and Helwig, 1965; Altschuler and Shake 1966), and Hermann (1911) reported an area of stratified epithelium, 3 mm long, near two prepyloric ulcers. Large areas of gastric leukoplakia, however, are very rare, and the following case is therefore reported.

### CASE REPORT

W.H.S., a male aged 56 years, had suffered from dyspepsia since 1967. A pyloroplasty was performed in 1971 during which no abnormalities were noted. The patient presented again in May 1975 with intermittent upper abdominal pain and vomiting, unrelieved by food or diet, and loss of weight of 12 Kg during the preceding 8 months. A barium meal showed a rounded mass, thought to be benign, in the gastric fundus; this was subsequently confirmed by gastroscopy. The upper half of the stomach was resected with an end-to-side oesophagogastrostomy. Recovery was uneventful and the patient has remained symptom-free since.

*Operation specimen:* This consisted of a short oesophageal stump together with sleeve of stomach, 17 x 9 cm. About 1.5 cm from the cardia there was a firm, well-defined, whitish-grey submucous mass shaped like three-quarters of a circle, measuring 6.5 x 5.5 x

**Fig. 1. Sleeve of stomach with central submucosal leiomyoma. Upper pole covered by smooth greyish epithelium with adjoining small ulcer. About ¾ of natural size.**

**Fig. 2. Thick layer of stratified epithelium with muscularis mucosae and submucosa (overlying the leiomyoma).**
**Haematoxylin and eosin. x 43**

**Fig. 3. Similar area but with slight diffuse chronic submucosal infiltration.**
**H.E. x 43**

3 cm, with a mucosal ulcer, 1 x 1 x 0.4 cm, on top. The rugose gastric mucosa between the oesophagus and the mural mass was greyish in appearance and was continuous with the lining of the mural tumour, the upper part of which was covered by taut, smooth, whitish leukoplakic epithelium for about 3.5 cm (fig. 1).

*Microscopical examination* (75/5345): The submucous tumour was a leiomyoma, and the presence of a shallow mucosal ulcer was confirmed. The gastric mucosa between oesophagus and mural neoplasm had been replaced by stratified epithelium which extended over the upper pole of the mass, forming a smooth, non-keratinizing squamous lining, from 0.1 to 2 mm thick (figs. 2 and 3). Very slight, patchy chronic inflammatory infiltration was present in the submucosa.

### DISCUSSION

Only five previously recorded cases of gastric leukoplakia have been traced, all but one in the older literature (Table). Leersum and Rotgans (1899) reported its occurrence in a woman aged 22 years who had been suffering from gastritis for 7 years; large numbers of squamous epithelial cells were present in the gastric washings. Gastric analysis failed to show any hydrochloric acid. Eventually she could tolerate only liquids, and a total gastrectomy was performed. The stomach had been transformed into a narrow tube admitting but one finger, with central peptic ulcer 3 x 1.5 cm. Its wall was 0.5 to 1.6 cm thick, and almost the entire gastric lining was leukoplakic, consisting microscopically of high stratified epithelium.

The patient described by Singer (1930) was a man aged 51 years who had been suffering from abdominal pain and vomiting for 3 years. Serological tests for syphilis were strongly positive. Radiological examination showed an hourglass stomach due to a central constriction. A subtotal gastrectomy was carried out; the resected specimen showed a markedly shrunken stomach, its wall varying from 0.9 to 1.5 cm thick. Apart from the central constriction, 2.5 cm long, the gastric lumen nowhere admitted more than 2 fingers. Below the stricture there was a diverticulum-like pouch, immediately above which there was a whitish quadrangular area of leukoplakia, 2 cm wide; microscopically this consisted of 15 to 20 rows of stratified epithelium. There was no evidence of a syphilitic infection.

Watson et al. (1936) reported a male aged 31 years with an hourglass stomach due to a central stricture which they regarded as tuberculous, but which would undoubtedly be regarded now as a manifestation of Crohn's disease. The stomach was resected and the upper part, above the stenosis, was found to be lined by stratified epithelium.

Sailer (1943) reported a negro woman who had become emaciated and anaemic after suffering for several years from intermittent attacks of abdominal pain and vomiting. Serological tests for syphilis were strongly positive. Free hydrochloric acid was not detected after histamine challenge. Radiological examination showed a small and contracted stomach with no signs of peristalsis. She subsequently died from a urinary infection, after a total illness of 5 years. At necropsy the stomach was found to be very small, with a markedly thickened wall and flattened mucosa. The pylorus was narrow but patent. Microscopically the gastric mucosa, from the cardia to 7 cm above the pylorus, had been replaced by stratified epithelium; the muscular coats were thickened, but there was no evidence of a syphilitic infection.

Carr and Squires (1962) reported a negro female aged 68 years with a 3-year history of lassitude and exertional dyspnoea. There was a marked macrocytic anaemia with a positive occult blood test. A barium meal showed a large gastric filling defect, partly obstructing the cardia. Laparotomy revealed a large mass apparently involving the entire stomach from oesophagus to pylorus; a subtotal gastrectomy was performed. Macroscopically the entire stomach was covered by leukoplakic, greyish-white, papillary mucosa, which extended through the wall, forming greyish-white verrucous nodules on the gastric serosa. Microscopically the stomach was lined by mature, papillary, hyperkeratotic squamous epithelium which, by invagination, had formed large submucous inclusions. The regional lymph nodes were not involved.

Squamous epithelium lining the stomach is a rare finding of uncertain histogenesis. It could be due to heterotopia, to metaplasia, or to downward extension of the oesophageal epithelium.

It is unlikely that heterotopia of the oesophageal epithelium could account for gastric leukoplakia. As already noted, such heterotopic islands in the gastric fundus are rare, and have hitherto all been of microscopical size. Furthermore, it must be considered more than a coincidence that focal leukoplakia, as observed by Sailer (1943) and in the present case, should be confined to the site of mural abnormalities.

Squamous metaplasia is often seen in the bronchial mucosa, and may also occur in nasal and cervical polyps, in the ducts of salivary glands and of the pancreas and, rarely, in endometrial glands (Haines and Taylor, 1975) and in the renal pelvis (Willis, 1958). In contrast, squamous metaplasia of the gastric mucosa is very rare. However, in cases of focal leukoplakia remote from the cardia, as described by Hermann (1911) and by Singer (1930), no other interpretation would seem to be adequate.

Extension of the oesophageal epithelium downwards appears to have occurred in most of the other cases recorded. In these patients there was obstruction to the passage of food, either by a mural tumour as in the present case, or a localized gastric constriction (Singer, 1930; Watson et al., 1936), or tube-like narrowing of the entire stomach of unknown aetiology (van Leersum and Rotgans, 1899; Sailer, 1943).

The case of squamous gastric papillomatosis described by Carr and Squires (1962) appears to be unique, resembling in some respects the "epithelioma cunniculatum" reported by Aird et al. (1954). It may, possibly, be akin to low-grade squamous-cell carcinomas at other sites, for example those arising in old osteomyelitic sinuses.

Mechanical factors thus seem to be important for the development of gastric leukoplakia, although direct squamous metaplasia may also occur occasionally. It is also possible that a dietary factor, such as vitamin-A deficiency (Sailer, 1943) may be a contributory cause.

CASES OF GASTRIC LEUKOPLAKIA

| *No.* | *Authors & Year* | *Patients' sex & age* | *Clinical symptoms* | *Length of history (years)* | *Stomach macroscopical* | *Leukoplakia* | *Microscopical features* | *Remarks* |
|---|---|---|---|---|---|---|---|---|
| 1 | van LEERSUM & ROTGANS 1899 | F 22 | dysphagia, taking fluids only | 7 | shrunken narrow tube | entire mucosa | stratified epithelium | numerous squamous epithelial cells in gastric washings; no free HCl acid |
| 2 | SINGER 1930 | M 51 | abdominal pain, vomiting, gastric stasis | 3 | shrunken, thick-walled hourglass | 2 cm square, above central constriction | stratified epithelium | diverticulum-like pouch below constriction and leukoplakic area (syphilis) |
| 3 | WATSON et al. 1936 | M 31 | epigastric pain, vomiting, loss of weight | 3/12 | shrunken, hourglass | upper half, above central constriction | stratified epithelium | Crohn's disease |
| 4 | SAILER 1943 | F 52 | abdominal pain, vomiting, anaemia, cachexia | 5 | shrunken, thick-walled | from cardia to 7 cm from pylorus | stratified epithelium | No free HCl acid (syphilis) |
| 5 | CARR & SQUIRES 1962 | F 68 | lassitude, nausea, indigestion | 3 | normal size | entire mucosa | invasive keratinizing squamous papillomatosis | positive occult blood test, macrocytic anaemia, postoperative death |
| 6 | PRESENT CASE | M 56 | epigastric pain, vomiting, loss of weight | 8 | normal size | 5 cm, fundal | stratified epithelium | fundal submucous leiomyoma, 6.5 x 3.5 x 3 cm. |

### REFERENCES

AIRD, I., JOHNSON, H.D., LENNOX, B. and STANSFIELD, A. G. 1954. Epithelioma cunniculatum. A variety of squamous carcinoma peculiar to the foot. *Brit. J. Surg.*, **42**, 245.

ALTSCHULER, J. H., and SHAKA, J. A. 1966. Squamous cell carcinoma of the stomach. Review of the literature and report of a case. *Cancer, Philad.*, **19**, 831, fig. 5.

BOSWELL, J. T., and HELWIG, E. B. 1965. Squamous cell carcinoma and adenoacanthoma of the stomach. A clinicopathologic study. *Cancer, Philad.*, **18**, 181, fig. 9.

CARR, G. L., and SQUIRES, Gretchen. 1962. Squamous papillomatosis of the stomach. A new pathologic entity: Report of a case. *Amer. J. Surg.*, **28**, 790.

HERMANN, A. 1911. Zur Frage der Epithelmetaplasie. *Wien. klin. Wchnschr.*, **24**, 168.

HAINES, M., and TAYLOR, C. W. 1975. Gynaecological Pathology, 2nd ed. Churchill Livingstone, *London*, pp. 171-172, fig. 6.15.

van LEERSUM, E. C., and ROTGANS, J. 1899. Extirpatie der geheele maag. Oesophago-duodenastomie. Endogastritis obliterans. De maaglooze meusch. *Nederl. Tijdschr. v. Geneesk.*, **35**, 993.

SAILER, S. 1943. Diffuse metaplastic gastritis in a patient with prolonged cachexia and macrocytic anaemia. *Arch. Path.*, **35**, 730.

SINGER, H. A. 1930. Leukoplakia of the stomach. *Arch. Path.*, **9**, 676.

STOUT, A. P., and LATTES, R. 1957. Tumors of the esophagus. Armed Forces Institute of Pathology, *Washington D.C.* Section V, Fascicle 20, p.13, figs. 3, 4 and 6.

WATSON G. W., FLINT, E. R., and STEWART, M. J. 1936. Hyperplastic tuberculosis of the stomach causing hour-glass deformity, with complete squamous metaplasia of the upper loculus. *Brit. J. Surg.*, **24**, 333.

WILLIS, R. A. 1958. The borderland of embryology and pathology. Butterworth, *London*, p.516, fig. 209.

## Figures and Tables

**Fig. 1. f1:**
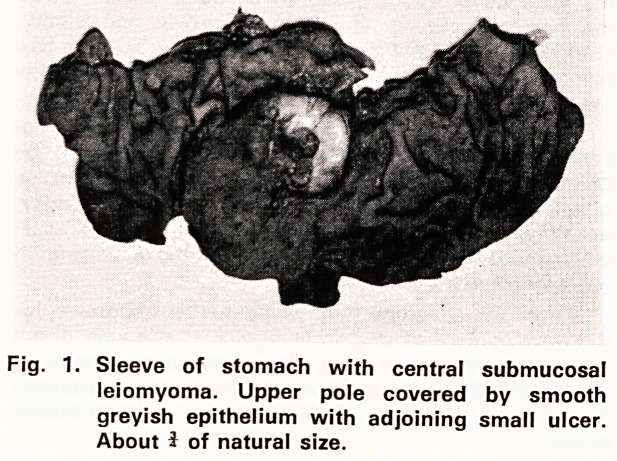


**Fig. 2. f2:**
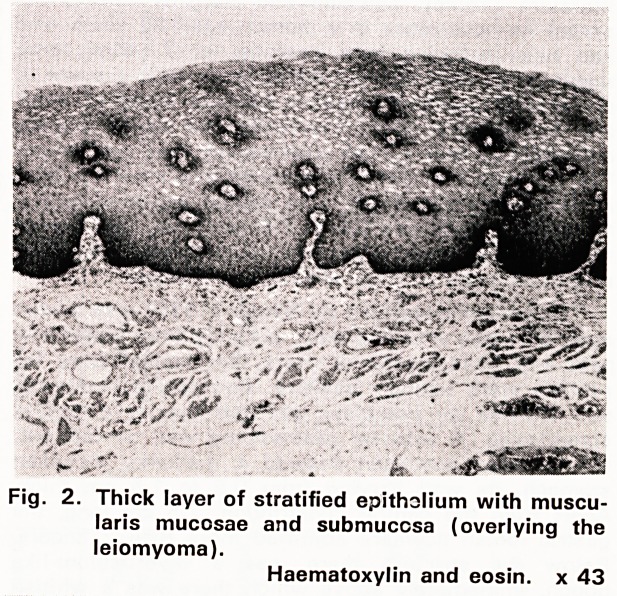


**Fig. 3. f3:**